# 55-Week Treatment of Mice with the *Unani* and *Ayurvedic* Medicine Pomegranate Flower Ameliorates Ageing-Associated Insulin Resistance and Skin Abnormalities

**DOI:** 10.1155/2012/350125

**Published:** 2011-12-28

**Authors:** Jianwei Wang, Xianglu Rong, Irene S. I. Um, Johji Yamahara, Yuhao Li

**Affiliations:** ^1^Division of Metabolism, Faculty of Basic Medical Sciences, Chongqing Medical University, 1 Yixueyuan Road, Yuzhong District, Chongqing 400016, China; ^2^Department of Pharmacology, Guangzhou University of Chinese Medicine, Guangzhou 510006, China; ^3^Disease State Management Group, Faculty of Pharmacy, The University of Sydney, NSW 2006, Australia; ^4^Pharmafood Institute, Kyoto 602-8136, Japan; ^5^Endocrinology and Metabolism Group, Faculty of Pharmacy, The University of Sydney, NSW 2006, Australia

## Abstract

PPARs play a pivotal role in regulating lipid and glucose homeostasis and are involved in diverse biological activities in skin. Pomegranate flower (PGF, an antidiabetic therapy in *Unani* and *Ayurvedic* medicines) has been previously demonstrated to activate both PPARalpha/gamma. Here, we found that treatment of mice with the diet containing PGF powder over 55 weeks attenuated ageing-induced abnormal increases in the homeostasis model assessment of insulin resistance, glucose concentrations during oral glucose tolerance test, and adipose insulin resistance index. The diet tended to decrease the excessive peri-ovary fat mass. It, however, increased the thinned subcutaneous fat thickness. In addition, the diet restored decreases in skin water content, epidermis thickness, and collagen density in corium. Thus, our results demonstrate that long-term treatment with the *Unani* and *Ayurvedic* therapy ameliorates ageing-induced insulin resistance, which is associated with reversal of ageing-induced fat redistribution. Further, PGF attenuates ageing-mediated undesirable skin abnormalities.

## 1. Introduction

Ageing is an important risk factor for most of the common diseases, such as type 2 diabetes and cardiovascular diseases. A fundamental process associated with ageing is dysregulation of energy homeostasis [1]. Advance in age is frequently associated with impaired glucose handling and a decline in glucose tolerance [1, 2]. Insulin plays a pivotal role in regulating glucose metabolism. Insulin resistance is the fundamental defect in type 2 diabetes and closely associated with obesity, dyslipidemia, and cardiovascular diseases [3]. On the other hand, the skin, one of the largest organs of the body, is affected frequently by the ageing process. Skin ageing leads to a direct reduction in water and collagen contents, thereby weakening skin functions [4]. 

Peroxisome proliferator-activated receptors (PPARs) are ligand-activated transcription factors belonging to the steroid nuclear receptor family. Three different isoforms (*α*, *β*/*δ*, and *γ*) have been identified and play a key role in the transcriptional regulation of genes responsible for the control of lipid and glucose metabolism [5]. PPAR*γ* and PPAR*α* are molecular targets for the insulin-sensitizers thiazolidinediones and the lipid-lowering drugs fibrates, respectively [6]. PPARs are also involved in various biological activities in epidermis, such as keratinocyte proliferation and differentiation, epidermal barrier maturation and recovery, sebocyte activity, and melanocyte differentiation [7]. Due to their diverse functions in skin biology, PPARs are also research targets for the understanding and treatment of many skin diseases.

*Punica granatum* Linn, commonly known as pomegranate, belongs to the Punicaceae family. Pomegranate flower (PGF) is an antidiabetic remedy in *Unani* and *Ayurvedic* medicines [8, 9]. In China, the flower has been also used in the treatment of grey hair in young men [10], suggesting that PGF may have antiageing property. PGF prominently contains polyphenols (321 ± 8 mg/g in the ethanolic extract, such as gallic acid and ellagic acid) and triterpenoids (oleanolic acid and ursolic acid) [11]. It has been demonstrated that PGF extract decreases blood glucose concentration in young diabetic animals [11–14].

We have recently identified PGF as a dual PPAR*α*/*γ* activator [11–13]. PGF extract and oleanolic acid were found to specifically enhance PPAR*α* luciferase reporter gene activity in human embryonic kidney 293 cells [13], while the extract and polyphenols gallic acid and ellagic acid activate PPAR*γ*-mediated gene expression and activity [12, 15, 16]. In addition, both oleanolic acid and ursolic acid have been shown to induce PPAR*α* expression in human skin cell line HaCaT [17, 18].

It has been suggested that some of the existing therapies for metabolic diseases might exert beneficial effects by tapping into the machinery that regulates ageing [1]. In contrast, the drugs targeting the mechanisms of ageing are inherently more valuable than drugs ameliorating only the disease by treating the symptoms [1]. We speculated that PGF, the herb with dual PPAR*α*/*γ* activator properties might meet this criterion. In this context, we in the present study investigated the effects of long-term treatment with PGF on ageing-associated metabolic abnormalities and skin changes in mice.

## 2. Materials and Methods

### 2.1. Pomegranate Flower (PGF)

PGF was collected in Maharashtra state, India. PGF was identified and characterized by HPLC to contain 0.82% gallic acid, 0.79% oleanolic acid, and 0.26% ursolic acid in methanolic extract [11–13]. Dried PGFs were grounded into a fine powder and mixed with a standard diet containing water 8.2%, crude protein 20.4%, crude fat 6.9%, ash 5.6%, fiber 1.7%, and soluble nonnitrate 57.2% (Oriental Yeast, Chiba, Japan) at the ratio of 0.25% PGF or 0.5% PGF (w/w).

### 2.2. Animals and Experimental Protocol

**Figure 1:**
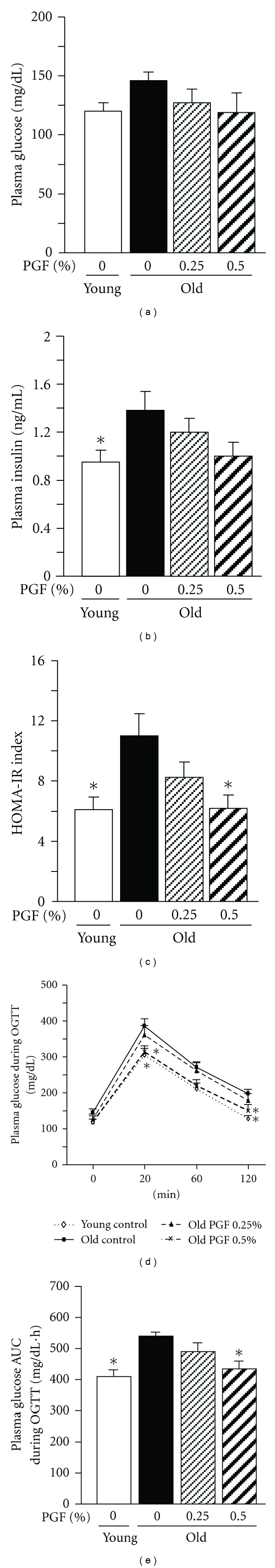
The effects of PGF powder on the fasted plasma glucose (a) and insulin (b) concentrations, the index of the homeostasis model assessment of insulin resistance (HOMA-IR) (c), and glucose concentrations (d) and AUC (e) during oral glucose tolerance testing (glucose dosage: 2 g/kg) in old female ddY mice. Animals consumed the diet containing 0.25%, 0.5% pomegranate flower (PGF), or a standard diet for 55 weeks. The corresponding young mice were used as young controls. Data are means ± SEM (*n* = 6 each group) *versus* old control, *P < 0.05.

All experimental procedures were carried out in accordance with the Guiding Principles for the Care and Use of Laboratory Animals approved by The Japanese Pharmacological Society [http://plaza.umin.ac.jp/JPS1927/jps/Animal.pdf]. Female ddY mice aged 4 weeks (Kiwa Laboratory Animals, Wakayama, Japan) were housed (3 mice per cage) in an air-conditioned room at  23 ± 1°C and 50–70% relative humidity with a 12-hour light/dark cycle. Mice were provided ad libitum with water and a standard diet. Animals were allowed free access to the food and water for 1 week before the experiments commenced. After one-week acclimation, 18 mice were divided into 3 groups (6 mice each group): old control, old 0.25% PGF, and old 0.5% PGF. Body weights were comparable between the groups before treatments commenced (Figure 1(c)). Mice in old 0.25% PGF group were fed the diet containing 0.25% PGF powder, while mice in old 0.5% PGF group were given the diet containing 0.5% PGF powder for 55 weeks. Animals in old control group were given the corresponding standard diet only. Food intake and body weight were determined at week 53. Additional 6 female ddY mice aged 5 weeks (after one-week acclimation) were used as young controls. Oral glucose tolerance tests (OGTT) were performed at week 54. Animals were weighed again at week 55 and then killed by prompt dislocation of the neck vertebra. Peri-ovary white adipose tissue was collected and weighed. Dorsal skin with subcutaneous fat tissue was also collected for water content determination and histological examination.

### 2.3. Oral Glucose Tolerance Test (OGTT)

Mice were fasted overnight with free access to water before OGTT. Mice received a glucose solution (2 g/kg) by gavage. Blood samples were collected prior to and 20, 60, and 120 min after administration of glucose solution for determination of plasma concentrations of glucose and/or insulin, total cholesterol, triglyceride and non-esterified fatty acids (NEFA). The homeostasis model assessment of insulin resistance (HOMA-IR) index was calculated according to the following formula: [insulin (*μ*IU/mL) × glucose (mM)]/22.5 [19, 20]. Glucose concentration area under curve (AUC) was calculated. Adipose insulin resistance (Adipo-IR) index was calculated as the following formula described previously [19]: [Adipo-IR = fasted insulin (mmol/L) × fasted NEFA (pmol/L)].

### 2.4. Blood Biochemical Determination

Plasma concentrations of glucose, total cholesterol, triglyceride, and NEFA were determined using commercial enzymatic methods (kits from Wako, Osaka, Japan). Plasma insulin concentrations were determined by ELISA (kit from Morinaga, Tokyo, Japan).

### 2.5. Determination of Skin Water Content

A portion of wet dorsal skin tissue (*≈*1 g) was weighed, minced, and dried in a tube at 80°C until a constant weight was obtained. Skin water content was calculated as g/g wet tissue.

### 2.6. Histological Examination

A portion of skin together with subcutaneous fat tissue was fixed with 10% formalin and embedded in paraffin. 4-micron sections were cut and stained with Mason's staining for examination of skin histology (IX-81, Olympus Corporation, Tokyo, Japan). The epidermis thickness, corium collagen density (histogram), and subcutaneous fat thickness were measured using an ImageJ 1.43 analyzing system. At least 100 measurements from 5 individual fields were performed for each animal.

### 2.7. Data Analysis

All results are expressed as means ± SEM. Data were analyzed by 1-factor analysis of variance (ANOVA). If a statistically significant effect was found, the Newman-Keuls test was performed to isolate the difference between the groups. *P* values less than 0.05 were considered as indicative of significance.

## 3. Results

### 3.1. Effects of PGF on Ageing-Associated Metabolic Variables in Mice

The fasted old animals appeared to increase in plasma glucose (Figure 1(a)) insulin concentrations (Figure 1(b)), and HOMA-IR index (Figure 1(c)). In the OGTT assessments, plasma glucose concentrations at 20 and 120 min after exogenous glucose stimulation (Figure 1(d)), as well as glucose AUC (Figure 1(e)), were also significantly higher (Figure 1(d)). Consumption of 0.5% PGF diet showed a trend to decrease the concentrations of fasted glucose and insulin. The most significant effects appeared to be on the decrease of HOMA-IR index and the inhibition of the abnormal increase in plasma glucose concentrations at 20 and 120 min, and glucose AUC in OGTT. Consumption of 0.25% PGF diet showed lesser effect on the plasma glucose and insulin concentrations, as well as HOMA-IR index.

**Figure 2:**
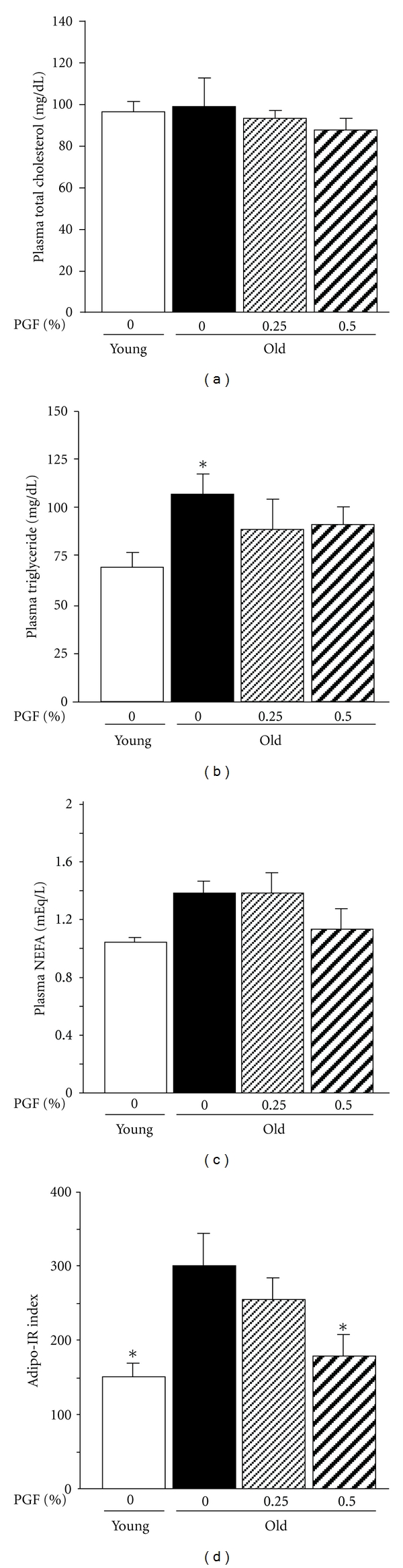
The effects of PGF powder on the fasted plasma concentrations of total cholesterol (a), triglyceride (b), nonesterified fatty acids (NEFA) (c), and adipose insulin resistance (Adipo-IR) index (d) in old female ddY mice. Animals consumed the diet containing 0.25%, 0.5% pomegranate flower (PGF) or a standard diet for 55 weeks. The corresponding young mice were used as young controls. Data are means ± SEM (*n* = 6 each group) *versus* old control, *P < 0.05.

Compared to the corresponding young controls, the old mice exhibited increased fasted plasma triglyceride concentrations (Figure 2(b)), whereas fasted plasma total cholesterol (Figure 2(a)) and NEFA concentrations (Figure 2(c)) were not significantly different between young and old controls. However, Adipo-IR index was significantly increased in old mice (Figure 2(d)). Consumption of 0.5% PGF diet decreased Adipo-IR index but did not significantly decrease plasma total cholesterol, triglyceride, and NEFA concentrations. 0.25% PGF diet did not significantly affect all these variables.

**Figure 3:**
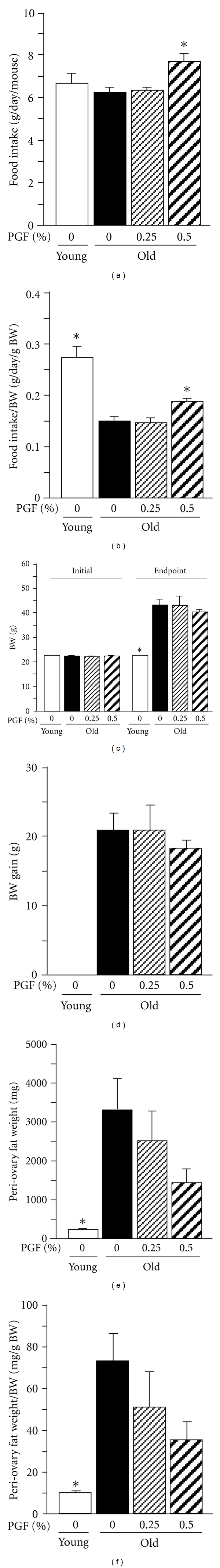
The effects of PGF powder on the average food intake (a), ratio of food intake to body weight (b), body weights (c), body weight gain (d), peri-ovary fat weight (e), and ratio of peri-ovary fat weight to body weight (f) in old female ddY mice. Animals consumed the diet containing 0.25%, 0.5% pomegranate flower (PGF), or a standard diet for 55 weeks. The corresponding young mice were used as young controls. Data are means ± SEM (*n* = 6 each group). BW, body weight; *versus* old control, *P < 0.05.

**Figure 4:**
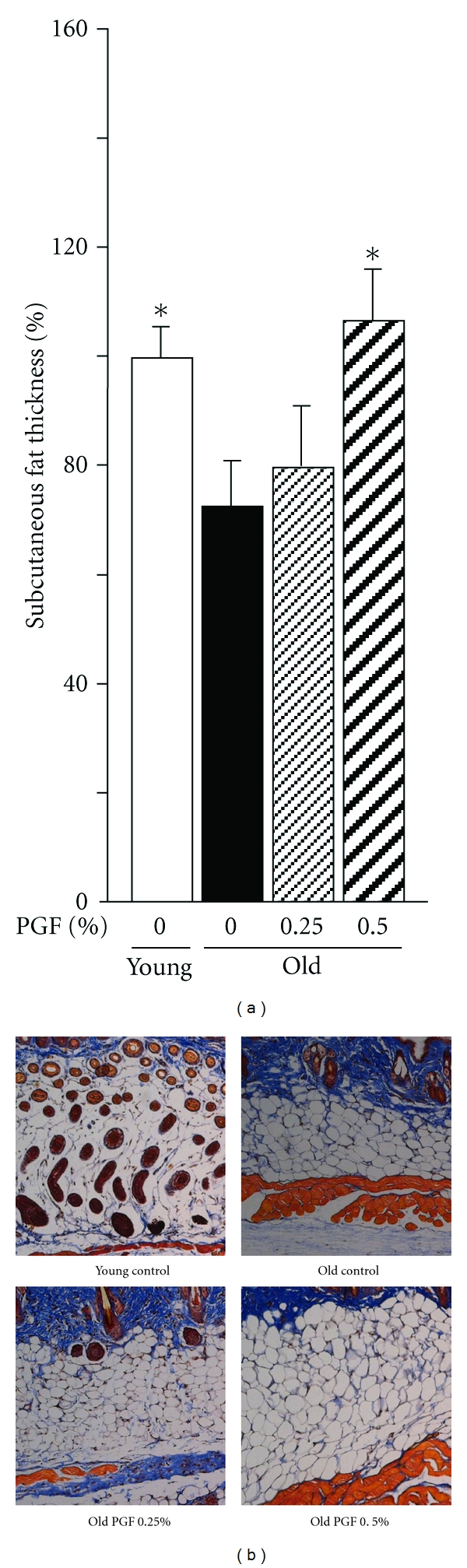
The effects of PGF powder on the subcutaneous fat thickness (a) and representative images showing histology of subcutaneous fat tissue (Mason's staining, ×100) (b) in old female ddY mice. Animals consumed the diet containing 0.25%, 0.5% pomegranate flower (PGF), or a standard diet for 55 weeks. The corresponding young mice were used as young controls. Data are means ± SEM (*n* = 6 each group) *versus* old control, *P < 0.05.

There was no difference in food intake between young and old controls (Figure 3(a)). However, the ratio of food intake to body weight was markedly decreased in old mice compared to young controls (Figure 3(b)). Body weight (Figure 3(c)), peri-ovary fat weight (Figure 3(e)), and the ratio of peri-ovary fat weight to body weight (Figure 3(f)) at the endpoint of the experiment were markedly increased in old controls compared to the corresponding young controls. In contrast, subcutaneous fat thickness (Figures 4(a) and 4(b)) were decreased by one-third in old mice compared to young controls. Treatment with 0.5% PGF diet significantly increased food intake (Figure 3(a)) and partially restored the decreased ratio of food intake to body weight (Figure 3(b)). This treatment also showed a trend to decrease peri-ovary fat weight (Figure 3(e)) and the ratio of peri-overy fat weight to body weight (Figure 3(f)), but did not affect body weight (Figure 3(c)) and body weight gain (Figure 3(d)). Furthermore, it significantly restored the decline of subcutaneous fat thickness (Figures 4(a) and 4(b)). 0.25% PGF diet showed minimal effect on all variables (Figures 3(a)–3(f) and 4(a)and 4(b)).

### 3.2. Effects of PGF on Ageing-Associated Skin Changes in Mice

**Figure 5:**
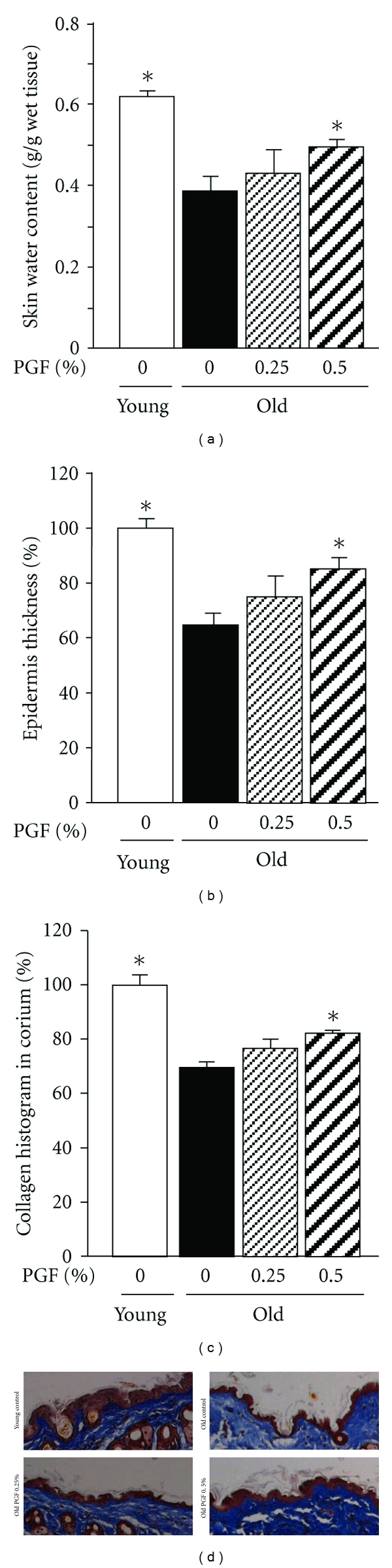
The effects of PGF powder on the skin water content (a), epidermis thickness (b), collagen histogram in corium (c), and representative images showing histology of epidermis and corium collagen density (Mason's staining, ×100) (d) in old female ddY mice. Animals consumed the diet containing 0.25%, 0.5% pomegranate flower (PGF), or a standard diet for 55 weeks. The corresponding young mice were used as young controls. Data are means ± SEM (*n* = 6 each group) *versus* old control, *P < 0.05.

The skin water content (Figure 5(a)), epidermis thickness (Figures 5(b) and 5(d)), and histogram of Mason's stained area in corium (reflecting the density of collagen accumulation) (Figures 5(c) and 5(d)) in old mice were decreased by one-third, compared to those of young controls. Consumption of 0.5% PDF diet significantly restored the declines of these variables, while 0.25% PDF diet showed minimal effect (Figures 5(a)–5(d)).

## 4. Discussion

In the present study, old mice showed an increase in HOMA-IR index and a decline in glucose clearance in the OGTT assessments, suggesting ageing-associated insulin resistance. Consumption of 0.5% PGF diet reduced the increased HOMA-IR index and inhibited the abnormal increase in glucose concentrations and AUC during OGTT in old mice. Thus, these results suggest that PGF ameliorates ageing-associated insulin resistance.

Obesity is a well-established metabolic risk factor. Increased adipose tissue mass, especially in the visceral compartment, represents one of the major risk factors for the development of type 2 diabetes [21]. An increase in visceral fat mass appears to be a risk factor for type 2 diabetes; by contrast, when majority of the fat is deposited subcutaneously and visceral fat mass is low, obese individuals are relatively healthy [22, 23]. Old individuals experience a progressive redistribution of fat from subcutaneous to visceral regions, making subcutaneous fat tissue thinner and abdominal fat tissue greater [24–26]. The increase in visceral fat and decrease in subcutaneous fat contributes to the insulin resistance that occurs in peripheral tissues [1, 27–29]. It has been demonstrated that reduction of plasma insulin concentrations by the PPAR*α* agonist fenofibrate is accompanied by a decrease in visceral fat mass in high-fat-diet-fed rats [7, 30] or in lipogenic, simple-carbohydrate-diet-fed mice [31]. In contrast, amelioration of insulin sensitivity by the PPAR*γ* agonists, pioglitazone, and rosiglitazone, is accompanied by an increase in subcutaneous fat mass, but not in visceral fat weight in obese rats [30, 32], and also in the patients with type 2 diabetes [33]. In the present study, old mice showed an increase in peri-ovary (visceral) fat mass and a decrease in subcutaneous fat thickness. This redistribution of fat was reversed after long-term consumption of PGF diet. Furthermore, the increased Adipo-IR index (indicating insulin sensitivity in adipose tissue, [19]) was also attenuated. Thus, it is likely that reversal of fat redistribution is involved in PGF consumption-elicited amelioration of ageing-associated insulin resistance. These positive consequences may be as a result of the dual PPAR*α*/*γ* activator properties of PGF.

The consumed food was similar in old mice to their corresponding young controls. Additionally, the ratio of food intake to body weight was much lower in old mice than their corresponding young controls. However, the body weights and peri-ovary fat mass were much higher in old mice. These results suggest an ageing-associated decrease in nutrition metabolism (energy expenditure). Interestingly, PGF treatment increased food intake and the ratio of food intake to body weight in old mice. Nevertheless, this treatment did not increase body weight. It has been demonstrated previously that the PPAR*γ* agonists rosiglitazone [32] and pioglitazone [30] increase food intake. Thus, the PPAR*γ* activator [13] property of PGF possibly contributes to the increased food intake.

PPAR*γ* is not expressed in mouse and human keratinocytes [34, 35]. In contrast, upregulation of PPAR*α* can be observed in the interfollicular epidermis upon proliferation by stimuli or hair plucking and during wound healing [34]. During epidermal differentiation, the expression of PPAR*α* increases, and stimulation of PPAR*α* increases the synthesis of cholesterol and ceramides in keratinocytes [36]. PPAR*α* activators profoundly influence lipid metabolism in reconstructed epidermis [37]. It has been demonstrated that oleanolic acid and ursolic acid, both of which are contained in PGF [11], induce the differentiation of human keratinocytes via a PPAR*α* pathway [17, 18]. Furthermore, treatment of hairless mice with these compounds increases epidermis thickness, enhances the recovery of epidermal permeability barrier function after disruption by tape stripping, and increases ceramide content and hydration levels in skin [17, 18]. The prescription of PGF for grey hair in Traditional Chinese Medicine [10] has suggested the potential of the herb in skin ageing. In the present study, ageing induced substantial decrease in skin water content, epidermis thickness, and collagen density in corium. PGF treatment attenuated these declines. These results demonstrate the benefit of PGF to skin ageing. PPAR*α* activation by PGF likely plays a role in these effects.

## 5. Conclusion

The present study demonstrates that the *Unani* and *Ayurvedic *medicine pomegranate flower ameliorates ageing-associated insulin resistance, which is associated with reversal of ageing-induced fat redistribution from subcutaneous to visceral regions. In addition, PGF attenuates ageing-mediated undesirable skin changes. This study provides potentially important results that may lead to further research, which supports and extends these findings to clinical trials.
